# Lay perspectives on the social and psychological functions of heroes

**DOI:** 10.3389/fpsyg.2015.00130

**Published:** 2015-02-17

**Authors:** Elaine L. Kinsella, Timothy D. Ritchie, Eric R. Igou

**Affiliations:** ^1^Department of Psychology and Centre for Social Issues Research, University of Limerick, LimerickIreland; ^2^Department of Psychology, Saint Xavier University, Chicago, ILUSA; ^3^Department of Psychology, University of Limerick, LimerickIreland

**Keywords:** hero, heroism, leader, role model, social and psychological functions, morality, altruism, person perception

## Abstract

Declaring and thinking about heroes are common human preoccupations but surprisingly aspects of heroism that reinforce these behaviors are not well-understood. In four thematically consistent studies, we attempt to identify lay perspectives about the psychological functions served by heroes. In Study 1, participants (*n* = 189) freely generated open-ended descriptions of hero functions, which were then sorted by independent coders into 14 categories (e.g., instill hope, guide others). In Study 2, in an attempt to identify the most important functions associated with heroes, participants (*n* = 249) rated how each function corresponded with their personal views about heroes. Results from a confirmatory factor analysis suggested that a three-factor model of hero functions fit the data well: participants thought that heroes enhanced the lives of others, promoted morals, and protected individuals from threats. In Study 3 (*n* = 242), participants rated heroes as more likely to fulfill a protecting function than either leaders or role models. In Studies 4A (*n* = 38) and 4B (*n* = 102), participants indicated that thinking about a hero (relative to a leader or an acquaintance) during psychological threat fulfilled personal enhancement, moral modeling, and protection needs. In all, these findings provide an empirical basis to spur additional research about the social and psychological functions that heroes offer.

## INTRODUCTION

Heroes have played an important role in society for centuries (Campbell, 1949) and their influence remains evident and prevalent in modern life (Zimbardo, 2007; Sullivan and Venter, 2010; Allison and Goethals, 2011, 2013a; Franco et al., 2011; Kinsella et al., 2015). Survey data from one recent sample revealed that 66% of the participants reported having a personal hero (Kinsella et al., 2010). This underscores the fact that heroism is a pervasive and everyday phenomenon. Unsurprisingly, it has been posited that heroes exert psychological influence on others (Sullivan and Venter, 2005). The variety of heroes that exist—whistle-blowers, martyrs, civil heroes, political heroes, and humanitarians (Zimbardo, 2007)—suggests the far-reaching utility of heroes. Yet, heroism has received relatively little attention in psychology (Becker and Eagly, 2004; Sullivan and Venter, 2005). Related topics such as generativity (e.g., Mansfield and McAdams, 1996), prosocial behavior (e.g., Hart and Fegley, 1995), whistleblowing (e.g., Lewis et al., 2014), and moral exemplars (e.g., Matsuba and Walker, 2005; Walker and Frimer, 2007; Frimer et al., 2011, 2012, 2013) are present in the literature and offer insights into persons who display some prototypical hero features. Few researchers, however, have considered why individuals have or want heroes (Goethals and Allison, 2012).

Empirical endeavors to understand heroes are gaining momentum (e.g., Allison and Goethals, 2011, 2013a; Franco et al., 2011; Goethals and Allison, 2012; Kinsella et al., 2015; Allison et al., unpublished). So far, many of these endeavors have progressed our understanding of what constitutes a hero in modern times; however, researchers have not yet explicitly theorized and empirically substantiated the array of social and psychological functions heroes might fulfill for individuals. A person who shows the prototypical hero features of bravery, sacrifice, conviction, risk-taking, and moral integrity for an honorable purpose (see Kinsella et al., 2015) is likely to provide psychological and social functions for individuals who encounter (or cogitate about) them. The focus of the present article is to systematically examine lay perspectives about the psychological and social functions provided by heroes. We believe that studying the psychological influence of heroes on individuals is a fascinating and worthy topic of study, especially given that heroes are often spatio-temporally distant (e.g., sometimes dead or remote). Focusing on understanding hero functions is likely to offer insights into the processes by which heroes influence individuals and help to discern ways to effectively harness the positive influence of heroes in education, healthcare, communities, or organizations. Examining possible functions fulfilled by heroes may provide another source of evidence about prototypical hero features (e.g., a hero described as providing an inspiring or uplifting function is likely to be characterized as inspirational), thus informing our understanding of the concept.

Understanding how people comprehend the social world can be enlightened by the ways people think about and infer meaning from what occurs around them (Heider, 1958). Increasingly, in health care settings, the lay conceptions explanatory model (Kleinman et al., 1976), is increasingly applied by medical professionals to gain critical insights into what is most important to the individual, what they believe about their health, and what they think will influence them psychologically. As research on attitudes, attitude and behavior, person perception (e.g., stereotyping), self-regulation, and metacognition has shown, people’s beliefs shape their reality and behavior (Heider, 1958; Kruglanski, 1975; Snyder, 1984; Dweck and Leggett, 1988; Igou, 2004; Fiske and Taylor, 2008). We adopt this perspective for investigating the topic of heroism. In order to understand how heroes are *used* in everyday life, it is important to examine how heroes are perceived, what qualifies as a hero, and how people think they can *use* them. Systematically identifying lay perspectives about a topic can be useful in helping to formulate common views that dominate thinking about a given psychological construct. Importantly, examining lay conceptions can be helpful for contributing to a conceptual framework for the development of explicit theories (Sternberg, 1985). In essence, our research makes an important first step toward understanding the social and psychological functions that heroes provide.

Existing literature typically focuses on one aspect of heroic influence, such as social control (Klapp, 1954), rescue from physical harm (Becker and Eagly, 2004), or symbolic immortality (Becker, 1973). In all, the result is a fragmented and diverse interpretation of the many possible functions that heroes may serve for groups and for individuals. This makes it difficult to develop a psychological theory of heroic influence. Before detailing four new empirical studies, we offer a synthesis of existing literary accounts of functions provided by heroes into three broad themes: enhancing, moral modeling, and protecting, which are briefly summarized below.

First, heroes are described in the literature as uplifting and enhancing the lives of others. Heroes may arouse positive emotions such as awe, gratitude, or admiration (Algoe and Haidt, 2009). People may experience positivity as result of being associated with their hero’s exceptional accomplishments (Allison and Goethals, 2011); this process is termed *basking in reflected glory* (Cialdini, 2007). Heroes may motivate individuals toward being a better person by raising awareness of ought or ideal selves (Klapp, 1969). Also, heroes have been described as directing our own ambitions away from “narrow, self-centered concerns” (Singer, 1991, p. 249). These type of encounters may trigger a period of world-focused savoring and social connectedness (*world focus*; Bryant and Veroff, 2007), evoking a sense of positive communion with nature and with others. Applying these ideas, The Heroic Imagination Project^1^ was set up to offer information about heroism that individuals may use to transform negative situations. Also, the Hero Construction Company^2^ uses inspiring narratives about heroes to promote heroic (rather than condemning bullying behavior) in schools. These projects use accounts of heroes such as Nelson Mandela, Rosa Parks, Daniel Ellsberg, and Irena Sendler to educate and inspire others toward create positive change.

Second, heroes are described as modeling morals and values. Heroes uphold the values of society (Carlyle, 1840), act as comparison targets for the masses (Pretzinger, 1976), and model virtues (Cohen, 1993). Also, heroes may help people to understand the norms and values within society (Erikson, 1977; Cohen, 1993). Heroes have been described as displaying moral integrity (Kinsella et al., 2015), doing the right thing (Schwartz and Schwartz, 2010), and showing a noble purpose without selfishness (Singer, 1991). Heroes prompt people to do what they can for those who need help, endorsing other-regard (Flescher, 2003). In fact, most heroes meet Colby and Damon’s (1992) criteria for serving as moral exemplars. It may not be realistic to emulate heroes that show moral fortitude, but the encounter may evoke a period of introspection which helps individuals to avoid moral complacency (Flescher, 2003).

Third, the etymology of the word heroes (from Greek *heros*) suggests that heroes protect others (Harper, 2010). Some philosophers and psychologists have alluded to the idea that heroes protect against threats to perceptions about one’s own meaning or purpose in life. For example, Hobbs (2010) suggested that heroes offer resources to adults who feel disillusioned. Heroes who uphold cultural values and norms may also serve as a resource for dealing with threats to uncertainty, meaning, or other existential dilemmas (Becker, 1973). Similarly, individuals often strive to create a meaningful life (Duckworth et al., 2005) based on society’s values, often modeled by heroes. Through such means, people create a lasting impact and achieve symbolic immortality (Goethals and Allison, 2012).

Based on our literature review, three broad categories of hero functions are accounted for: enhancing, moral modeling, and protecting. To reach consensus about the types of social and psychological functions that heroes provide, we suggest that examining lay conceptions about hero functions is a useful precursor to developing a theory of hero functions. As such, we first attempted to distill the range of functions that people associate with heroes, and then synthesized this information into meaningful categories (Studies 1 and 2). Second, we illustrated the extent to which individuals perceived that heroes influenced others in a similar or distinct ways to other persons of influence (Study 3). Third, we examined the extent to which people perceived benefits from thinking about heroes, leaders (Study 4A), or acquaintances (Study 4B) during times of threat or unfulfilled needs (e.g., low self-esteem, social isolation, uncertainty) as predicted by Klapp (1969) and Becker (1973). Thus, the present article responds to the call for further research on heroes (Zimbardo, 2007; Franco et al., 2011) and particularly to the call for further research on what good that heroes might do for people (Allison and Goethals, 2011).

## STUDY 1

The study of the impact of persons’ lay theories on their social understanding has a long history in personality and social psychology (e.g., Hong et al., 2001). Following in that tradition, Study 1 aimed to systematically analyze lay persons’ responses to the question: “In your view, what functions do heroes serve?” The term *functions* was adopted in order to facilitate participants’ inclusion of both positive and negative assessments of heroic actors. The resulting exemplars were analyzed systematically, in accordance with prototype methods (Hassebrauck, 1997). We expected that the most representative functions provided by heroes would be those that our participants expressed most frequently.

### METHOD

#### Participants

One-hundred and eighty-nine participants (116 women, 73 men, *M*_age_ 29.98 years, SD_age_ = 11.88, age range: 18–73 years) were recruited via Facebook^TM^ and snowball sampling via email (*n* = 164), and in the local city center (*n* = 25). Participants originated from North America (*n* = 90), Europe (*n* = 89), and Australasia or Africa (*n* = 10). Gender frequencies by geographical location were as follows American (59% female), European (65% female), and Australasian or African (56% female). The mean ages of participants in each geographical location was as follows: American (*M* = 28, SD = 11.10), European (*M* = 32, SD = 12.89), and Australasian or African (*M* = 32, SD = 8.80).

#### Materials and procedure

Ethical approval was obtained from the University of Limerick’s Research Ethics Committee (Studies 1–4). Informed consent was obtained from all participants (Studies 1–4). Participants completed standardized materials either on paper or online. Those who completed the questionnaire online did not receive any compensation for their participation. Those who filled out the questionnaire in the city center received a coffee as a token of appreciation. Participants were asked: “In your view, what functions do heroes serve?” Participants were informed that “There are no correct or incorrect answers, and this is not a psychological test.” Responses were not timed. Participants were then thanked and debriefed (Studies 1–4).

### RESULTS AND DISCUSSION

A verbatim list of exemplars (*n* = 344) was compiled. An exemplar is defined as one item from a list, or one unit of meaning (Joffe and Yardley, 2004) from responses that contained multiple connected descriptions of hero functions.

During Phase 1 of coding, two research assistants sorted the original exemplars into superordinate thematic categories without prior knowledge about our predictions. This was achieved by grouping (a) identical exemplars, (b) semantically related exemplars (e.g., “give people hope” and “instill hope”), and (c) meaning-related exemplars into categories (e.g., “keep people safe” and “protect people from evil”) in accordance with the approach taken by previous research (Hepper et al., 2011). In the first round, the first coder identified 13 categories and the second coder identified 14 categories. To reach full agreement it was necessary to create a new category. The first coder’s category, *to inspire and motivate*, was split into two categories (i.e., *to inspire*, *to motivate)*, resulting in 14 function categories.

During Phase 2 of coding, the third and fourth coders independently matched each original exemplar (e.g., “helping somebody to pave the way toward a personal goal”) with the 14 categories (e.g., to help) identified by the first and second coders. There was 76% consistency between the third coder’s ratings and the original coding. There was 67% consistency between the fourth coder’s ratings and the original coding. Most of the inconsistencies arose where coders placed exemplars such as “builders of self-esteem,” “punish the bad,” and “they epitomize what we should be” in multiple categories. If we take semantic units that were multiply classifiable as confirmation of reliability, the figures rise to 83 and 87% which are comparable with other published articles (e.g., Gregg et al., 2008).

#### Categories of hero functions

The independent coders identified 14 categories of functions provided by heroes from the original 344 exemplars (see **Table 1**). The categories of functions that were identified are as follows: to help, to inspire, to motivate, to save, to be a role model, to protect, to instill hope, to improve morale and camaraderie, to make the world a better place, to do what no one else will, to remind people about the good in the world, to guide, to show morals and values, and to act against evil or danger. On average, participants described two exemplars (*M* = 2.05, SD = 1.30)^3^.

**Table 1:** Fourteen hero functions and relatedness ratings in Study 2.

Function	*M*	SD
To make the world better	6.48	1.44
To do what no one else will	6.47	1.61
To help	6.46	1.35
To instill hope	6.37	1.42
To be a role model	6.37	1.62
To protect	6.29	1.46
To save	6.28	1.56
To inspire	6.23	1.48
Acts against evil or danger	6.04	1.87
To motivate	5.98	1.60
To show morals and values	5.90	1.70
To guide	5.83	1.71
To improve morale	5.82	1.63
To remind people about good	5.65	1.84

*n* = 249. Heroic functions listed in order of Study 2 ratings, scale ranged from 1 (*not at all related to heroes*) to 8 (*extremely related to heroes*).

#### Linguistic analysis of hero functions

To provide additional information about the exemplars, all responses were subjected to analysis using the textual analysis software, Linguistic Inquiry and Word Count Version 2007 for Windows (LIWC; Pennebaker et al., 2007). LIWC compares each word from every participant’s response against an internal dictionary that contains English words, and then, reports a percentage of words that represent a psychological theme. For example, one participant wrote that heroes “remind us of the human potential,” and LIWC flagged the word human as belonging to the social theme. On average, participants’ descriptions consisted of 26% social (e.g., people, others), 20% affect (e.g., happy, positive), 19% positive emotion (e.g., love), 17% cognitive mechanism (e.g., ought, know), and 8% achievement (e.g., earn, win) themes. This is consistent with the view that heroic benefits are described in positive ways, in particular, relating to social topics, emotions, attitude formation, and taking action to pursue goals.

Some heroes were described as enhancing positive feelings about the self and others (to inspire, to motivate, increase morale) and modeling morals (to provide morals and values, to remind people of the good in the world). Other heroes were described as protecting people, either physically (e.g., “saving lives”) or emotionally (e.g., “to help people in a situation where they are in distress or despair and they are almost ready to give up”). These findings present empirical support to some ideas about why people *need* heroes presented by Allison and Goethals (2013b). For instance, those authors suggested that heroes give people hope and offer nurturance (enhancing); educate people about right and wrong, and validate our moral worldviews (moral modeling); and, save us when we are in trouble, pick us up when we are down, and deliver justice (protecting). Each are consistent with the three themes that we identified in the literature.

## STUDY 2

Participants were invited to rate the relatedness of each heroic function (identified in Study 1) to their own view of heroes. Researchers have used similar methods to identify exemplar representativeness of a prototype (e.g., Hepper et al., 2011). Based on the themes that emerged from the literature and from an exploratory factor analysis (EFA)^4^, we expected that the ratings of some functions would cluster together into three categories, with each factor a latent construct representing hero functions: *enhancing*, *moral modeling,* and *protecting*. We tested the extent to which a three-factor model fit the data via a confirmatory factor analysis (CFA).

**Table 2:** Factor loadings from factor analysis based on ratings in Study 2.

Functions	Enhancing	Protecting	Moral modeling
To motivate	**0.87**	0.03	-0.12
To be a role model	**0.82**	-0.04	-0.05
To inspire	**0.73**	-0.13	0.19
To instill hope	**0.57**	0.07	0.18
To improve morale	**0.52**	0.11	0.28
To guide	**0.41**	0.30	0.05
To save	-0.13	**0.96**	-0.12
To protect	0.08	**0.84**	0.03
To help	0.04	**0.74**	0.06
Acts against evil and danger	-0.18	**0.60**	0.25
To do what no one else will	0.24	**0.37**	0.03
To remind people about good	0.05	-0.03	**0.84**
To show morals and values	0.04	0.16	**0.57**
To make the world better	0.18	0.14	**0.44**

Bold indicates the high factor loadings for each hero function.

### METHOD

#### Participants

Two-hundred and forty-nine participants were recruited for this study in a local city center, on the University of Limerick campus, and via the psychological research website, http://psych.hanover.edu/ (120 women, 129 men, *M*_age_ = 32.64 years, SD_age_ = 12.48, age range: 18–67 years).

#### Materials and procedure

We offered the participants who we recruited on campus or in the local city center chocolate for their participation in the study. Participants recruited online were not compensated. Participants rated how closely each of the 14 functions of heroes related to their personal view of heroes. After each function category, some common exemplars were provided in brackets: “Inspiration (make you dream, show people what is possible, remind us of the human potential)” and “Shows morals and values (give us a set of values, conserve morals, and values).” All ratings were indicated on a Likert scale that ranged from 1 (not at all related) to 8 (extremely related). Readability statistics for the functions of heroes and associated exemplars include the Flesch Reading Ease = 67.6% and Flesch–Kincaid Grade Level 8.

### RESULTS AND DISCUSSION

#### Descriptive statistics

The ratings for hero functions ranged from 5.65 (to remind people about the good in the world) to 6.48 (to make the world better), on an 8-point Likert scale (see **Table 1**). These results support the idea that these 14 functions represent some of the most important functions provided by heroes.

#### Confirmatory factor analysis

A CFA tested the three-factor structure that was predicted from our analysis of the literature and from our preliminary results that emerged from an EFA. The analyses were conducted with LISREL 8.8.

In the CFA model, to save, to protect, to help, to do what no one else will, and to act against evil or danger were each specified as the latent factor *protecting*. To motivate, to role model, to inspire, to instill hope, to provide morale, and to guide were specified as the latent factor *enhancing*. Finally, to remind people about the good in the world, to show morals and values, and to make the world better were specified as the latent variable *moral modeling*. Results confirm that this three-factor model fit acceptably with the data, χ^2^(74, *n* = 248) = 232.82, *p* < 0.05, goodness of fit index (GFI) = 0.89, the non-normed fit index (NNFI) = 0.92, comparative fit index (CFI) = 0.94, root mean square error of approximation (RMSEA) = 0.08, and standardized root mean residual (SRMR) = 0.08. Bentler and Bonett (1980) recommended that measurement models have GFI, NNFI, and CFI of at least 0.90. According to Browne and Cudeck (1993), RMSEA between 0.05 and 0.08 represents a reasonably close fit, and, RMSEA > 0.10 represents an unacceptable model. Also, Hu and Bentler (1998) suggested that SRMR larger than 0.08 represents an unacceptable model fit.

In accordance with the variety of our participants’ responses, the data suggest that heroes provide more than a single, overarching psychosocial function. Indeed, a one-factor model fit the data inadequately, χ^2^(77, *n* = 248) = 584.73, *p* < 0.05, GFI = 0.70, NNFI = 0.81, CFI = 0.19, RMSEA = 0.19, and SRMR = 0.11. None of the fit statistics for the one-factor model reached 0.90 and the RMSEA was well above 0.10. We predicted three categories of heroic influence based on a review of the literature and our EFA results; indeed, the data suggest that this model fit the data well.

## STUDY 3

Leaders are typically described as persons who are responsible for organizing a group of people to achieve a common goal. More specifically, transformational leaders have been described as those who inspire others and create a future vision (Bass, 1990). Previous research suggests that transformational leaders may provide psychological functions to their followers (Ilies et al., 2005). Leaders are sometimes considered heroic. Allison and Goethals (2011, 2013a) draw attention to the number of leaders who are represented on their lists of popular heroes. Some hero functions could also describe the influence of leaders. We wondered if lay theories about hero functions would be measurably distinct from those of leaders.

Next, role models have been described as influential people who are often geographically close, similar in age, and share comparable experiences to their supporter (Brownhill, 2010). In 1991, Singer explained that role models who are closer to their follower are observed carefully and mimicked. Role models have previously been found to engage followers in prosocial behavior (Bryan and Test, 1967) and inspire others (Lockwood and Kunda, 1997). The words hero and role model are often used interchangeably. Thus, we wondered if lay theories about hero functions are measurably distinct from those of role models.

Given the etymology of the word *hero* (meaning ‘protector’), we expect that heroes would be the best protectors of psychological and physical well-being. Hence, Study 3 examines whether participants would rate the 14 functions (generated in Studies 1–2) equally for heroes, leaders, and role models.

### METHOD

#### Participants

Two-hundred and forty-two post-graduate students (136 females, 106 males, *M*_age_ = 30.60 years, SD_age_ = 10.64, age range: 18–66 years) were recruited for this online study via the University of Limerick intranet.

#### Materials and procedure

The study employed a between-groups design. Participants completed an online questionnaire that prompted them to bring to mind either a leader (*n* = 73), a role model (*n* = 95), or a heroic individual (*n* = 74). Persons were randomly distributed across conditions. Participants rated how closely each of the 14 functions of heroes (described in Studies 1 and 2) related to their personal view of heroes. After each function category, some common exemplars were provided in brackets: “Inspiration (make you dream, show people what is possible, remind us of the human potential)” and “Shows morals and values (give us a set of values, conserve morals and values).” All ratings were indicated on a Likert scale that ranged from 1 (not at all related) to 8 (extremely related).

### RESULTS AND DISCUSSION

#### Rating heroes, leaders, and role models on 14 hero functions

A multivariate General Linear Model evidenced a significant association between type of influential person and associated functions, Wilk’s Lambda *F*(28,452) = 2.48, *p* < 0.001, $\eta_{p}^{2}$ = 0.13. Univariate tests shows significant relationships between type of individual and ratings for the following (see **Table 3**): to help, to save, to motivate, to make the world better, to guide, and to do what no one else will do. Participants rated heroes as more likely to help, to save, to protect, to make the world better, and to do what no one else will. They rated leaders as more likely to motivate and to guide.

**Table 3:** Mean (SD) and inferential statistics tests that evidenced significant differences between type of influential person and the participants’ ratings of each in Study 3.

Function	Hero	Leader	Role model	*t*- and *p*-values	Effect size (*d*)
To help	6.05 (1.80)	5.01 (1.90)	–	3.38***	0.56
To save	5.84 (1.85)	–	5.02 (1.90)	2.95**	0.44
To save	5.84 (1.85)	4.95 (1.72)	–	3.20**	0.50
To do what no one else will	6.76 (1.81)	–	6.07 (1.92)	2.50**	0.37
Improve morale	6.71 (1.45)	–	6.19 (1.88)	1.98*	0.31
To make the world better	6.97 (1.55)	–	6.21 (2.03)	2.75**	0.42
To guide	6.55 (1.78)	7.16 (1.28)	–	-2.82**	0.39
To motivate	6.73 (1.53)	7.40 (0.89)	–	-3.30***	0.54

*p < 0.05; **p < 0.01; ***p < 0.001.

#### Rating heroes, leaders, and role models on categories of hero functions

Each heroic function was coded as belonging to one of the three categories from Study 2: protecting, enhancing, and moral modeling. A multivariate General Linear Model revealed an association between the type of influential person and the categories of hero functions, Wilk’s Lambda *F*(6,494) = 3.07, *p* < 0.01, $\eta_{p}^{2}$ = 0.04. Univariate tests indicated that there were significant relationships between type of individual and ratings for protecting. For instance, heroes were rated as more likely to save, to help, and to do what no one else will do.

There was a significant difference between ratings of *protecting* for heroes, leaders, and role models, *F*(2,249) = 4.07, *p* = 0.02, $\eta_{p}^{2}$ = 0.32. The pairwise comparison revealed mean differences between heroes (*M* = 6.09, SD = 1.46) and role models (*M* = 5.60, SD = 1.56), *t*(175) = 2.17, *p* = 0.03, *d* = 0.68. Further, the mean differences between heroes (*M* = 6.09, SD = 1.46) and leaders (*M* = 5.40. SD = 1.50) was significant, *t*(151) = 2.77, *p* = 0.01, *d* = 0.33.

The data highlight some important conceptual distinctions between persons of influence. Heroes, role models, and leaders have potential to serve both *enhancing* and *moral modeling* functions. Heroes may provide a *protecting* function beyond that of role models or leaders. Overall, heroes are more likely to help, save, protect, make the world better, and do what no-one else will than leaders or role models.

The findings illustrate that leaders were rated as more likely to guide and motivate than heroes or role models. This is probably not surprising given that political leaders such as Mandela and Mahatma Gandhi are considered heroic by millions of people and are famous for their ability to guide and motivate others. Leaders who display prototypical features of heroism may influence people in different ways than other leaders. For example, transformational leaders are defined as leaders who raise followers to higher levels of effort by appealing to their morals and values (Chmiel, 2000). Also, Allison and Goethals (2013a) helpfully point out that the distinction between indirect and direct leaders (e.g., Gardner, 1995) may help us to further understand the overlap between the concepts of hero and leader.

Participants in Study 3 most likely brought to mind direct leaders (e.g., Barack Obama, Angela Merkel), rather than indirect leaders (e.g., Helen Keller, Wesley Autrey). Thus, this study is most likely comparing heroes with direct leaders. Conceptual clarification is needed in order to tease apart the possible functions of direct and indirect leaders, and the overlap with heroic actors.

Role models, due to their accessibility to their follower, are often scrutinized in detail and mimicked (Singer, 1991). Whereas, heroes tend to be distant figures who have endured tremendous suffering and sacrifice for purposes of great nobility, whom we would not wish to emulate (Singer, 1991). These ideas are reflected in recent research that suggests that role models are generally physically close, from the same generation, and have comparable experiences to the follower (Brownhill, 2010).

Previous research has found that lay persons tend to think of role models as more *talented, honest, personable, exceptional*, and *humble* than heroes or leaders (Kinsella et al., 2015). Researchers have found that altruistic role models increase the likelihood that those around them engage in prosocial behavior (Bryan and Test, 1967). This is consistent with the findings here that role models provide a moral modeling function. Also, Lockwood and Kunda (1997) described the enhancing function of role models which is consistent with the present research. Of course, negative or ‘bad’ role models are unlikely to be a positive influence on others.

## STUDIES 4A AND 4B

In Studies 4A and 4B we examined the extent to which participants indicate that heroes, leaders and acquaintances fulfill enhancing, moral modeling, and protecting functions when experiencing social or psychological threats. We hypothesized that participants would consistently indicate that heroes fulfill the enhancing, moral modeling, and protecting functions to a greater extent than a leader or an acquaintance.

### PILOT

In a pilot study conducted on the University of Limerick campus, we asked participants (*n* = 42) to state whether they believed Nelson Mandela (former President of South Africa), Enda Kenny (Taoiseach, Leader of Fine Gael in Ireland) and Michael O’Leary (Chief Executive of RyanAir airlines) to be either a hero or a leader. Sixty-seven percent of our participants believed that Mandela is a hero rather than a leader or neither (i.e., non-hero/non-leader), in comparison with 64% who believed that Enda Kenny is a leader, and 67% who indicated that Michael O’Leary is a leader. In a study that we conducted in Kinsella et al. (2010), we found that Mandela was one of the most frequent heroes mentioned. Therefore, in Study 4A we used these target persons to examine perceived functions fulfilled by heroes and leaders in an Irish sample.

### METHOD

#### Participants and design

In Study 4A (within-subjects design), 38 participants (18 men, 20 women, *M*_age_ = 22.53, SD_age_ = 2.02) were asked to rate three persons of influence in three different scenarios (enhancing, moral modeling and protecting conditions). In Study 4B (mixed design), 102 participants (55 men, 47 women, *M*_age_ = 26.34, SD_age_ = 11.58) were randomly assigned to the enhancing, moral modeling, protecting, or control conditions, and then asked to rate both target persons (hero, acquaintance). Participants were recruited in the local city center and did not receive any compensation.

#### Procedure and materials

In Study 4A, participants were asked to read three statements representing the enhancing, moral modeling and protecting functions of heroes. For enhancing, participants read “If I felt negative about myself and others, thinking about (see person below) would increase my positive feelings about myself and other people, and motivate me to further develop my potential.” For moral modeling, participants read “If I felt disconnected from others and unmotivated to act for the good of the group, thinking about (see person below) would remind me of morals, values and ethics, and encourage me to behave in ways that benefit others.” For protecting, participants read “If I felt threatened in some way or worried about the future, thinking about (see person below) would increase my feeling of protection and safety, and help me to cope with uncertainty.” Participants were then requested to indicate how much they agreed with these three statements, in relation to three named targets (i.e., Nelson Mandela, Enda Kenny, and Michael O’Leary) on the rating scale provided (1 = *strongly disagree*, 7 = *strongly agree*).

In Study 4B, participants were assigned to one of four conditions: enhancing, moral modeling, protecting, and control. To rule out the possibility of a valence effect, we included a control condition that refers to more mundane social interactions (i.e., talking about the weather). This condition was included to control for the potential effect that heroes, positively represented targets, are generally rated more positively than others (i.e., valence effect), or whether heroes are rated more positively only on hero functions. Participants rated self-generated heroes and acquaintances. Specifically, participants were asked to write the name or initials of either a person in their life who they know slightly, but who is not a friend (i.e., an acquaintance), read a statement relating to one of the four conditions, and rate their responses on a 7-point Likert scale (1 = *strongly disagree*, 7 = *strongly agree*). On a separate page, participants were asked to write the names or initials of their personal hero, read a statement and rate their responses on the 7-point Likert scale. In Study 4B, the acquaintance (i.e., non-hero) is the main reference point. Crucially, we predicted that heroes would be viewed more positively than acquaintances at providing enhancing, moral modeling, and protecting functions; further, we expected no differences between targets in the control condition.

Participants in both studies rated specific targets, rather than abstract ideas, of heroes, leaders, and acquaintances. The enhancing, moral modeling, and protecting statements used in Study 4B were identical to those used in Study 4A. A control condition was included in Study 4B to reduce the possibility that heroes received higher ratings across all dependent social measures. As such, the control condition stated “If you think about the weather and how strongly you feel about it, can you see yourself having the wish to talk about it with __.” Discussing the weather in social settings is a prevalent norm in Ireland which forms the basis of relatively mundane social interactions. We use this control condition to examine whether heroes receive inflated ratings across all positive conditions.

### RESULTS

#### Enhancing condition

In Study 4A, for enhancing, there were statistically significant differences between the mean ratings for Mandela (*M* = 5.51, SD = 1.21), O’Leary (*M* = 3.24, SD = 1.53) and Kenny (*M* = 2.89, SD = 1.58), Wilk’s Lambda = 0.478, *F*(2,35) = 25.59, *p* < 0.001, $\eta_{p}^{2}$ = 0.59. Paired samples t-tests were used to compare ratings for each of the target persons. There was a significant difference between mean ratings for Mandela and O’Leary, *t*(36) = 6.02, *p* < 0.001, *d* = 2.01 and for Mandela and Kenny, *t*(36) = 7.00, *p* < 0.001, *d* = 2.33 but not for the leaders, O’Leary and Kenny, *t*(36) = 1.17, *p* = 0.09, *d* = 0.39. Finally, in Study 4B, in the enhancing condition (*n* = 25), there was a statistically significant difference on ratings for acquaintance (*M* = 3.84, SD = 1.78) and for hero (*M* = 4.92, SD = 1.63), *t*(24) = –2.52, *p* = 0.02, *d* = 1.03.

#### Moral modeling condition

In Study 4A, for moral modeling, there were statistically significant differences between ratings for Mandela (*M* = 5.6, SD = 1.36), O’Leary (*M* = 2.68, SD = 1.75) and Kenny (*M* = 2.51, SD = 1.43), Wilk’s Lambda = 0.221, *F*(2,35) = 61.78, *p* < 0.001, $\eta_{p}^{2}$ = 0.78. There was a significant difference between mean ratings for Mandela and O’Leary, *t*(36) = 8.50, *p* < 0.001, *d* = 2.83, and for Mandela and Kenny, *t*(36) = 11.25, *p* < 0.001, *d* = 3.75. However, there was no significant difference for ratings between the leaders, O’Leary and Kenny, *t*(36) = –0.67, *p* = 0.51, *d* = 0.22. Finally, in Study 4B, in the moral modeling condition (*n* = 27), there was a statistically significant difference between acquaintance (*M* = 3.59, SD = 1.87) and for hero (*M* = 5.74, SD = 1.70), *t*(26) = –4.45, *p* < 0.001, *d* = 1.75.

#### Protecting condition

In Study 4A, for protecting, there were statistically significant differences between ratings for Mandela (*M* = 4.70, SD = 1.83), O’Leary (*M* = 2.62, SD = 1.53) and Kenny (*M* = 2.65, SD = 1.57), Wilk’s Lambda = 4.78, *F*(2,35) = 19.12, *p* < 0.001, $\eta_{p}^{2}$ = 0.52. There were significant differences between mean ratings for Mandela and O’Leary, *t*(36) = 6.27, *p* < 0.001, *d* = 2.09, and for Mandela and Kenny, *t*(36) = 5.19, *p* < 0.001, *d* = 1.73. However, there was no statistically significant difference on ratings for the leaders, O’Leary and Kenny, *t*(36) = –0.13, *p* = 0.90, *d* = 0.04. Next, in Study 4B, in the Protect condition (*n* = 26), there was a significance difference between acquaintance (*M* = 3.08, SD = 1.50) and hero (*M* = 5.38, SD = 1.86), *t*(25) = –5.34, *p* < 0.001.

#### Control condition

In Study 4B, as predicted, there were no reliable differences between heroes (*M* = 4.67, SD = 2.12) and acquaintances (*M* = 4.21, SD = 1.87) in the control condition, *t*(23) = 1.14, *p* = 0.27.

#### Interaction analyses for Study 4B

The findings from Studies 4A and 4B supported the hypotheses that participants reported that heroes (to a greater extent than leaders or non-hero targets) provide enhancing, moral modeling and protecting functions if a particular need is threatened or unfulfilled. To further examine this data, we created a heroic function variable comprising of an aggregate of the enhancing, moral modeling and protecting conditions. The non-heroic function variable represents the control condition.

Overall, heroes (*M* = 5.36, SD = 1.74) were rated by participants as more likely to provide a heroic function than acquaintances (*M* = 3.50, SD = 1.73). A mixed ANOVA was conducted for target person (hero and acquaintance) and functions (hero functions or non-heroic function), with repeated measures on the target person variable. There was a significant interaction between type of function provided and the target person associated with that function, *F*(1,100) = 7.10, *p* < 0.001, $\eta_{p}^{2}$ = 0.07. Participants who thought about a personal hero while imagining social psychological stress expressed greater fulfillment for hero functions than thinking of an acquaintance. Participants who thought about a personal hero while imagining a need to talk socially about the weather (control condition), showed no significant effect. There was a significant main effect for target person, Wilk’s Lambda = 0.84, *F*(1,100) = 19.42, *p* < 0.001, $\eta_{p}^{2}$ = 0.16. There was no significant main effect for functions, *F*(1,100) = 0.98, *p* = 0.98, $\eta_{p}^{2}$ = 0.

### DISCUSSION

In Study 4, two studies elucidated lay beliefs about the functions of heroes and in particular, how individuals may use heroes as a resource if a given need is threatened or unfulfilled. Participants rated heroes as more likely to fulfill enhancing, moral modeling, and protecting functions than other targets, offering support to our hypotheses. Study 4B illustrated that participants did not rate heroes higher across all positive social functions. Study 4B replicates and extends the findings from Study 4A. We think that participants were discerning in their beliefs that heroes serve enhancing, moral modeling, and protecting needs, but not necessarily other social or emotional needs (e.g., daily social pleasantries). In sum, we demonstrated that participants view heroes as a resource for coping when psychological or social needs are threatened or unfulfilled.

## GENERAL DISCUSSION

A primary goal of this research was to clarify lay perspectives about hero functions and to ascertain the extent to which such functions are similar to or different from each other, and to the themes that we identified in the exiting literature. This review led us to the assertion that the subjective functions provided by heroes can be represented in three categories: enhancing, moral modeling, and protecting.

Independent coder analyses of lay conceptions (Study 1) revealed 14 perceived functions provided by heroes, for example, to inspire, to protect, to guide, to instill hope, and to motivate. Another sample rated each of the 14 function categories in terms of importance (Study 2). CFA established that our predicted three-factor model, including the factors protecting, enhancing, and moral modeling, fit the data well in comparison to a poorly fitting one-factor model. In Study 3 we asked participants to rate heroes, role models, or leaders across all 14 hero functions. The results illustrated that heroes were perceived as more likely to help, to save, to protect, to make the world better, and to do what no one else will. Heroes were perceived by participants as protecting others more than both leaders and role models. In Studies 4A and 4B the results evidenced that participants viewed heroes as a resource for experiencing enhancement, moral modeling, and protection when psychological or social needs were threatened or unfulfilled. The present studies suggest that lay theories can provide a useful assessment in the study of heroism. We use the information from the literature and lay conceptions of heroes to form a conceptual framework, the Hero Functions Framework, which is integrative and can serve as a basis for future research. We describe this framework below.

### THE HERO FUNCTIONS FRAMEWORK

#### Enhancing function

According to lay conceptions, heroes motivate, act as a role model, inspire, instill hope, improve morale and camaraderie, and guide others. Participants described feeling positive affect when thinking of heroes, “making them feel happy” and “helping people to live a happy life.” Heroes were frequently described by participants as making people “feel better about the world,” “more positive about humanity,” and reminding people of “the good in the world.” To us, this makes sense, because when a person feels good about the self they are more positive and less misanthropic toward other people too (e.g., Ybarra, 1999). One person described heroes as “builders of self-esteem.” Heroes were portrayed as elevating and motivating people, for example, “[they] elevate the rest of us to a place of courage” or “elevate the consciousness of others.” The enhancing function is linked to previous writings about heroes who instigate periods of transcendence (Klapp, 1969), induce a perspective shift (Allison and Goethals, 2011), increase the positive emotions experienced by others (Algoe and Haidt, 2009), and increase social connectedness (Smith, 1976). Future research will help to clarify the apparent role of heroes in helping individuals to cope with or transcend difficult situations.

Upward social comparisons with role models (Lockwood and Kunda, 1997) and do-gooders (Minson and Monin, 2012) can sometimes result in perceived self-threats and self-deflation. Individuals do, however, sometimes actively seek out upward social comparisons in order to gain an accurate self-assessment and to self-enhance (Collins, 1996). In fact, a person can consciously prevent upward comparisons from influencing their self-evaluations and choose to use that information to inspire, motivate, and promote positive affect instead (Taylor and Lobel, 1989).

When experiencing the threat of uncertainty (e.g., during major life transitions), superior others and role models can be perceived as inspiring if the more established person has successfully overcome similar adjustment difficulties and their behaviors are perceived as attainable (Lockwood et al., 2012). The mystery behind heroes is that, although their exceptional behavior is normally out of reach of regular people and even though they are single exemplars which are particularly likely to induce judgmental contract effects, heroes still appear to produce motivational assimilation effects. We suspect this is because heroes, though individuals, embody abstract values. We believe that people typically process information about heroes at an abstract level and use the information as a source of motivation for their goals. Future research on heroes could draw from construal level theory (Trope and Liberman, 2010) to investigate the role of psychological distance on the social comparison interpretations of heroic influence.

Alternatively, the positive (and non-threatening) influence of heroes could be interpreted from a recent theory of inspiration. For instance, Thrash et al. (2010) note that people first appreciate the exceptional efforts of the inspirational target (resulting in feelings of transcendence and meaning) which in turn is translated into a personal desire to perform at a higher level in one’s own life (evoking feelings of self-responsibility and volitional control). In all, theories of social comparison and inspiration both help to generate specific hypotheses about heroes. Taken together, these ideas pave the foundation for future research into the psychological processes associated with the enhancing influence of heroes.

#### Moral modeling function

Some hero functions are abstract and symbolic, for example, reminding people about the good in the world, showing morals and values, and making the world a better place. Research about moral exemplars may elucidate the moral modeling function of heroes (Colby and Damon, 1992; Matsuba and Walker, 2005; Walker and Frimer, 2007; Frimer et al., 2011, 2012). In our studies, lay persons described heroes as “increasing positive feelings about humanity” and promoting “confidence that there is good in the world.” When a person feels good about their own self they are more receptive to negative information about themselves (Trope and Neter, 1994). Given this, it is no coincidence that heroes boost our feelings of happiness and simultaneously reveal our missing qualities.

Fascinatingly, participants described heroes as “moral symbols to protect everyday innocent people,” “providing moral goals for society,” and that they “personify the things we cannot articulate.” In our studies it was clear that some heroes were perceived by participants to act as agents of social justice, striving to improve the situations of the disadvantaged. This is consistent with Sorel (1912) who argued that social movements require a narrative with sufficient moral and emotional force to give clarity and inspiration to an account of events. Indeed, heroic individuals can give meaning to collective action and promote group solidarity. Narrative psychology offers a useful lens through which researchers and individuals can seek to understand the role of heroes in moral narratives.

Lay conceptions refer to heroes that make them “aware of the rest of humanity,” perhaps shifting their focus away from individual concerns and redirecting toward a world-focus perspective (Bryant and Veroff, 2007). This is consistent with previous research that suggests that moral exemplars typically integrate both agentic and communal motives (Frimer et al., 2011, 2012). In our research, one participant described how heroes teach us that it is possible to be altruistic in an egocentric world [similar to scholarly points made by Flescher (2003)], regulating the self toward more noble purposes (Singer, 1991), even when those decisions may require courage, conviction, and integrity. The extent that heroes influence moral willpower and moral decision-making, perhaps via a process of self-regulation, has not yet been investigated.

#### Protecting function

Lay conceptions suggest that heroes provide a protecting function: they save, help, guide, protect, act against evil or danger, and do what no one else will do. Heroes may help people to restore positive feeling about others and buffer negative feelings about themselves. For instance, one participant described a hero who helped her in a car crash. Another participant wrote about a hero who assisted her “to get through the tough times,” offering additional coping resources (suggested by Hobbs, 2010).

Heroes were frequently depicted as representing the “fight for good against evil” or “stopping the bad in humanity.” Those who believe that heroes are proactively taking action to combat evil or danger may feel safeguarded (e.g., “a hero’s job is making citizens feel safe”) and more certain about the future (e.g., “tomorrow we will be safe”). Other scholarly work indicates that persons use metaphors, myths, or symbols to give coherence to their lives (Campbell, 1988; Lakoff and Johnson, 2003). Perhaps heroes, similar to powerful myths and metaphors, are used as tools for dealing with uncertainty (Van den Bos, 2009). Both leaders and heroes were described as offering guidance and leadership through the complexity of daily life. This is interesting given that many heroes do not occupy formal leadership positions. Formal and informal leadership theory (Gardner, 1995) may help to elucidate the influence of heroes who occupy direct or indirect leadership positions (Allison and Goethals, 2013a). Traditionally, direct leaders pull a group toward a tangible goal, whereas indirect leaders (and heroes) guide a new way of thinking, being, or doing within a particular group, sometimes without tangible outcomes. This point underscores the value of current efforts to unveil the complexity of lay perspectives about the psychosocial functions fulfilled by heroes.

### CONTRIBUTION AND LIMITATIONS

Writers have alluded to the psychological benefits derived from heroic encounters, yet this fragmented information has not been synthesized or empirically studied. Until this point, the functions of heroes have been dealt with in a relatively superficial and piecemeal manner. Thus, the present research aimed to narrow the gaps in our understanding of heroes by presenting four studies that elucidate lay perspectives about the social and psychological functions of heroes. Similarly, we synthesize ideas about heroes in the extant literature, in an attempt to offer a novel conceptual framework, the Hero Functions Framework. With this framework in place, researchers can systematically assess the influence of heroes while simultaneously taking into account the type of hero, individual differences, and situational influences. Our research is a starting point, an important step in understanding how heroes are used psychologically and socially.

Klapp (1969) suggested that the media capitalize on the desire for heroes and present heroes (and more often pseudo-heroes) in order to fulfill this need and “vainly do we make scores of artificial celebrities grow where nature planted only a single hero” (Boorstin, 1992, p. 76). Other authors similarly noted that “the need for heroes is so strong that the media will manufacture pseudo-heroes to meet it” (Schwartz and Schwartz, 2010, p. 32). The impact of pseudo-heroism, celebrity culture, and negative role models is of serious concern for parents, educators, governments, researchers, and many others. For instance, a great deal of debate exists about the over-sexualization of children and teenagers as a result of exposure to negative role models and the absence of real heroes who help others to move toward more noble purposes (Singer, 1991). If people need external reference points for goals, standards, and ways to behave (Schlenker et al., 2008), it is important to make salient heroes, role models, and leaders who serve as models for desirable conduct in a particular group. We study heroes empirically with the hope that this information will be used in responsible ways that benefit others, albeit not heroically but with good intensions. Unfortunately the great tyrants of history have been held up as heroes by the unsuspecting masses, skillfully manipulated through propaganda. Part of the value of this research may be in deterring inappropriate hero worship as much as encouraging appropriate hero worship.

So far, we have examined lay conceptions of heroes—perceivable and conceivable functions expressed by hundreds of mostly young adults—rather than actual or measurable functions that heroes fulfill. It is possible that lay persons overstate the psychosocial functions that heroes provide in their everyday lives, or that heroes provide functions which are outside of their conscious awareness. In view of the introspective illusion (e.g., Pronin, 2009), one might question whether and to what extent people, if they are not experts on their own mental processes, can provide valid reports about how heroes function psychologically. Although, lay theories about mental processes can be accurate (see Nisbett and Wilson, 1977), we acknowledge that the present research offers suggestive evidence only; it is part of a relatively new empirical story and impetus for further research.

### FUTURE RESEARCH

Future research needs to examine how lay perspectives relate to actual changes in the self and self-regulatory processes. The next phase of this research will be to demonstrate the effects of information about heroes on participants in lab settings. Specifically, there is a need to examine the *protecting, enhancing, and moral modeling functions* of heroes as dependent variables affected by exposure to heroes of heroic acts. This is a broader research question than we intended to study in the present article.

So far, the functions listed for ‘known’ versus ‘unknown’ heroes have not been independently assessed. People’s relationship with their heroes varies widely and as a result they may derive different benefits from encounters. For instance, it is likely that people who have a personal relationship with their heroic grandmother will derive different benefits than a person who has developed a parasocial relationship (Horton and Wohl, 1956) with Nelson Mandela. The types of parasocial relationships people have with influential people, such as heroes, celebrities, or sports stars, are underexplored.

Heroes have been described as shaping and representing culture (Hegel, 1975) and providing a source of social control (Klapp, 1954). The heroes worshipped in a given group may reveal that a group’s most cherished values. In some cases, heroes represent minority values, speaking out against dominant cultural values, and as agents of change. In the present article, a full analysis of cultural differences in lay perceptions about heroes was not possible. The few participants from Africa, Australia, and Asia preclude us to make generalizations across countries or continents. Nonetheless, we think that studying the variety of cultural representations of heroes is a fruitful avenue for future research. For instance, research suggests that Japanese individuals tend to cherish the suffering of their heroes (Benedict, 1946); whereas, in Western cultures, there is a tendency to savor heroic efforts that result in a happy outcome (Heine et al., 1999). Such research looms on the horizon in our labs.

## CONCLUSION

The present research studies potential social and psychological functions served by heroes using deductive and inductive methods. Our research offers a conceptual framework that facilitates the development of a psychological theory of heroism, as well as helping to pave the way for additional research on hero functions and the consideration of how gender and culture might each influence and be influenced by heroes. Given the assortment of physical, psychological, and social reward people associate with heroes, it is unsurprising that many individuals offer “homage, commemoration, celebration, and veneration” to their heroes in return (Klapp, 1954, p. 57).

## Conflict of Interest Statement

The authors declare that the research was conducted in the absence of any commercial or financial relationships that could be construed as a potential conflict of interest.

## Footnotes

1 http://heroicimagination.org/

2 http://www.theherocc.com/

3 There was no significant sex differences between the number of exemplars reported, *t*(187) = –1.01, *p* = 0.31. There was no relationship between age and number of exemplars reported (*r* = 0.07, *p* = 0.36). There were no significant differences between USA and European participants regarding the number of exemplars provided, *t* < 1. There were no significant differences between community and online participants regarding the number of exemplars provided, *t* < 1.

4 To reduce the number of categories of functions and summarize the patterns of correlations among the functions, EFA was conducted. An initial principal component’s analysis revealed three factors with Eigenvalues over Kaiser’s criterion of one and in combination explained 65% of the variance. The scree plot also depicted three factors. We subjected the ratings of the 14 functions to an analysis involving principal axis factoring with oblique rotation (Promax). The results from the factor correlation matrix show that all correlations between factors are 0.32 or above, suggesting there is enough variance to warrant oblique rotation (Tabachnick and Fidell, 2007). The results of the EFA (see **Table 2**) suggested three factors that represent our respondents’ ratings of hero functions. The ratings that loaded onto Factor 1 included to save, to protect, to help, to do what no one else will, and to act against evil or danger. We termed this factor *protecting*. The items that loaded strongly onto Factor 2 were to motivate, to role model, to inspire, to instill hope, to provide morale, and to guide. We call this factor *enhancing*. The items that loaded onto Factor 3 were to remind people about the good in the world, to show morals and values, and to make the world better. We named this factor *moral modeling*.

## References

[B1] Algoe S. Haidt J. (2009). Witnessing excellence in action: the other-praising emotions of elevation, admiration, and gratitude. *J. Posit. Psychol.* 4 105–127 10.1080/17439760802650519 19495425 PMC2689844

[B2] Allison S. T. Goethals G. R. (2011). *Heroes: What They Do and Why We Need Them*. New York, NY: Oxford University Press.

[B3] Allison S. T. Goethals G. R. (2013a). *Heroic Leadership.* New York, NY: Routledge.

[B4] Allison S. T. Goethals G. R. (2013b). *10 Reasons Why We Need Heroes.* Available at: http://blog.richmond.edu/heroes/ [accessed May 17, 2013].

[B5] Bass B. M. (1990). From transactional to transformational leadership: learning to share the vision. *Organ. Dyn.* 18 19–32 10.1016/0090-2616(90)90061-S

[B6] Becker E. (1973). *The Denial of Death.* New York, NY: Simon & Schuster.

[B7] Becker S. W. Eagly A. H. (2004). The heroism of men and women. *Am. Psychol.* 59 163–178 10.1037/0003-066X.59.3.163 15222859

[B8] Benedict R. (1946). *The Chrysanthemum and the Sword.* Boston, MA: Houghton Miﬄin.

[B9] Bentler P. M. Bonett D. G. (1980). Significance tests and goodness of fit in the analysis of covariance structures. *Psychol. Bull.* 88 588–606 10.1037/0033-2909.88.3.588

[B10] Boorstin D. J. (1992). *The Image: A Guide to Pseudo-Events in America*. New York, NY: Vintage Books.

[B11] Browne M. W. Cudeck R. (1993). “Alternative ways of assessing model fit,” in *Testing Structural Equation Models* eds Bollen K. A. Long J. S. (Newbury Park, CA: Sage) 136–162.

[B12] Brownhill S. (2010). The ‘brave’ man in the early years (0–8): the ambiguities of the role model. *Paper presented at British Educational Research Association* University of Warwick UK.

[B13] Bryan J. H. Test M. A. (1967). Models and helping: naturalistic studies in aiding behavior. *J. Pers. Soc. Psychol.* 6 400–407 10.1037/h0024826 6082466

[B14] Bryant F. B. Veroff J. (2007). *Savoring: a New Model of Positive Experience*. Mahwah, NJ: Lawrence Erlbaum Associates.

[B15] Campbell J. (1949). *The Hero with a Thousand Faces.* Princeton, NJ: Princeton University Press.

[B16] Campbell J. (1988). *The Power of Myth.* New York, NY: Doubleday.

[B17] Carlyle T. (1840). *On Heroes, Hero-Worship, and the Heroic in History*. London: Chapman and Hall (Reprinted by The Echo Library in 2007).

[B18] Chmiel N. (2000). *Introduction to Work and Organizational Psychology: A European Perspective*. Oxford: Wiley-Blackwell.

[B19] Cialdini R. B. (2007). *Influence: The Psychology of Persuasion*. New York, NY: Collins.

[B20] Cohen S. (1993). For parents particularly: lessons in moral behavior: a few heroes. *Child. Educ.* 68 168–170 10.1080/00094056.1993.10520921

[B21] Colby A. Damon W. (1992). *Some do Care: Contemporary Lives of Moral Commitment*. New York, NY: Free Press.

[B22] Collins R. L. (1996). For better or worse: the impact of upward social comparison on self-evaluations. *Psychol. Bull.* 119 56–69 10.1037/0033-2909.119.1.51

[B23] Duckworth A. L. Steen T. A. Seligman M. E. P. (2005). Positive psychology in clinical practice. *Ann. Rev. Clin. Psychol.* 1 629–651 10.1146/annurev.clinpsy.1.102803.144154 17716102

[B24] Dweck C. S. Leggett E. L. (1988). A social-cognitive approach to motivation and personality. *Psychol. Rev.* 25 109–116.

[B25] Erikson E. H. (1977). *Toys and Reasons: Stages in the Ritualization of Experience*. London: Marion Boyars.

[B26] Fiske S. T. Taylor S. E. (2008). *Social Cognition: From Brains to Culture*. Boston, MA: McGraw-Hill.

[B27] Flescher A. M. (2003). *Heroes, Saints, and Ordinary Morality*. Washington, DC: Georgetown University Press.

[B28] Franco Z. Blau K. Zimbardo P. (2011). Heroism: a conceptual analysis and differentiation between heroic action and altruism. *Rev. Gen. Psychol.* 5 99–113 10.1037/a0022672

[B29] Frimer J. A. Biesanz J. C. Walker L. W. MacKinlay C. W. (2013). Liberals and conservatives rely on common moral foundations when making moral judgements about influential people. *J. Pers. Soc. Psychol.* 104 1040–1059 10.1037/a0032277 23586414

[B30] Frimer J. A. Walker L. J. Dunlop W. L. Lee B. H. Riches A. (2011). The integration of agency and communion in moral personality: evidence of self-interest. *J. Pers. Soc. Psychol.* 101 149–163 10.1037/a0023780 21574724

[B31] Frimer J. A. Walker L. J. Lee B. H. Riches A. Dunlop W. L. (2012). Hierarchical integration of agency and communion: a study of influential moral figures. *J. Pers.* 80 1117–1145 10.1111/j.1467-6494.2012.00764.x 22224747

[B32] Gardner H. (1995). *Leading Minds: An Anatomy of Leadership*. London, UK: Harper Collins.

[B33] Goethals G. R. Allison S. T. (2012). Making heroes: the construction of courage, competence and virtue. *Adv. Exp. Soc. Psychol.* 46 183–235 10.1016/B978-0-12-394281-4.00004-0

[B34] Gregg A. P. Hart C. M. Sedikides C. Kumashiro M. (2008). Everyday conceptions of modesty: a prototype analysis. *Personal. Soc. Psychol. Bull.* 34 978–992 10.1177/0146167208316734 18463395

[B35] Harper D. (2010). *Online Etymology Dictionary.* Available at: http://www.etymonline.com

[B36] Hart D. Fegley S. (1995). Prosocial behavior and caring in adolescence: relations to self-understanding and social judgement. *Child Dev.* 66 1346–1359 10.2307/1131651 7555220

[B37] Hassebrauck M. (1997). Cognitions of relationship quality: a prototype analysis of their structure and consequences. *Personal. Relationsh.* 4 163–186 10.1111/j.1475-6811.1997.tb00137.x

[B38] Hegel G. W. F. (1975). *Aesthetics: Lectures on Fine Art*. Oxford: Clarendon Press.

[B39] Heider F. (1958). *The Psychology of Interpersonal Relations.* New York: Wiley.

[B40] Heine S. J. Lehman D. R. Markus H. R. Kitayama S. (1999). Is there a universal need for positive self-regard? *Psychol. Rev.* 106 766–794 10.1037/0033-295X.106.4.766 10560328

[B41] Hepper E. G. Ritchie T. D. Sedikides C. Wildschut T. (2011). Odyssey’s end: lay conceptions of nostalgia reflect its original Homeric meaning. *Emotion* 12 102–119 10.1037/a0025167 21859192

[B42] Hobbs A. (2010). “Heroes and heroism,” in *James Cook (Producer), Free thinking festival* ed. Cook J. (Gateshead: BBC Radio 3).

[B43] Hong Y. Levy S. R. Chiu C. (2001). The contribution of lay theories approach to the study of groups. *Pers. Soc. Psychol. Rev.* 5 98–106 10.1207/S15327957PSPR0502-1

[B44] Horton D. Wohl R. R. (1956). Mass communication and para-social interaction: observations on intimacy at a distance. *Psychiatry* 19 215–229. 13359569 10.1080/00332747.1956.11023049

[B45] Hu L. Bentler P. M. (1998). Fit indices in covariance structure modelling: sensitivity to underparameterized model misspecification. *Psychol. Methods* 3 424–453 10.1037/1082-989X.3.4.424

[B46] Igou E. R. (2004). Lay theories in affective forecasting: the progression of affect. *J. Exp. Soc. Psychol.* 40 528–534 10.1016/j.jesp.2003.09.004

[B47] Ilies R. Morgeson F. P. Nahrgang J. D. (2005). Authentic leadership and eudaemonic well-being: understanding leader-follower outcomes. *Leadersh. Q.* 16 373–394 10.1016/j.leaqua.2005.03.002

[B48] Joffe H. Yardley L. (2004). “Content and thematic analysis,” in *Research Methods for Clinical and Health Psychology* eds Marks D. F. Yardley L. (London: Sage) 56–68.

[B49] Kinsella E. L. Ritchie T. D. Igou E. R. (2010). “Essential features and psychological functions of heroes,” in *Poster session presented at the Northern Ireland British Psychological Society Annual Conference* Enniskillen.

[B50] Kinsella E. L. Ritchie T. D. Igou E. R. (2015). Zeroing in on heroes: a prototype analysis of hero features. *J. Pers. Soc. Psychol.* 108 114–127 10.1037/a0038463 25603370

[B51] Klapp O. E. (1954). Heroes, villains, and fools, as agents of social control. *Am. Sociol. Rev.* 19 56–62 10.2307/2088173

[B52] Klapp O. E. (1969). *Collective Search for Identity.* New York, NY: Holt, Rinehart, and Winston.

[B53] Kleinman A. Eisenberg L. Good B. (1976). Culture, illness, and care: clinical lessons from anthropologic and cross-cultural research. *Ann. Intern. Med.* 88 251–258 10.7326/0003-4819-88-2-251 626456

[B54] Kruglanski A. H. (1975). The endogenous-exogenous partition in attribution theory. *Psychol. Rev.* 82 387–406 10.1037/0033-295X.82.6.387

[B55] Lakoff G. Johnson M. (2003). *Metaphors We Live* 2nd Edn. Chicago, IL: aUniversity of Chicago Press 10.7208/chicago/9780226470993.001.0001

[B56] Lewis D. Brown A. J. Moberly R. (2014). “Whistleblowing, its importance and the state of the research,” in *International Handbook on Whistleblowing Research* eds Brown A. J. Lewis D. Moberly R. Vandekerckhove W. (Cheltenham: Edward Elgar Publishing Limited).

[B57] Lockwood P. Kunda Z. (1997). Superstars and me: predicting the impact of role models on the self. *J. Pers. Soc. Psychol.* 73 91–103 10.1037/0022-3514.73.1.91

[B58] Lockwood P. Shaughnessy S. C. Fortune J. L. Tong M. (2012). Social comparisons in novel situations: finding inspiration during life transitions. *Personal. Soc. Psychol. Bull.* 38 985–996 10.1177/0146167212447234 22825208

[B59] Mansfield E. D. McAdams D. (1996). Generativity and themes of agency and communion in adult autobiography. *Personal. Soc. Psychol. Bull.* 22 721–731 10.1177/0146167296227006

[B60] Matsuba M. K. Walker L. W. (2005). Young adults moral exemplars: the making of self through stories. *J. Res. Adolesc.* 15 275–297 10.1111/j.1532-7795.2005.00097.x

[B61] Minson J. A. Monin B. (2012). Do-Gooder derogation: disparaging morally motivated minorities to defuse anticipated reproach. *Soc. Psychol. Personal. Sci.* 3 200–207 10.1177/1948550611415695

[B62] Nisbett R. E. Wilson T. D. (1977). Telling more than we can know: verbal reports on mental processes. *Psychol. Rev.* 84 231–259 10.1037/0033-295X.84.3.231

[B63] Pennebaker J. W. Francis M. E. Booth R. J. (2007). *Linguistic Inquiry and Word Count (LIWC): LIWC 2007*. Mahwah, NJ: Lawrence Erlbaum Associates.

[B64] Pretzinger K. (1976). The American hero: yesterday and today. *Humboldt J. Soc. Relat.* 4 36–40.

[B65] Pronin E. (2009). The introspection illusion. *Adv. Exp. Soc. Psychol.* 41 1–55 10.1016/S0065-2601(08)00401-2

[B66] Schlenker B. R. Weigold M. F. Schlenker K. A. (2008). What makes a hero? The impact of integrity on admiration and interpersonal judgment. *J. Pers.* 76 323–355 10.1111/j.1467-6494.2007.00488.x 18331277

[B67] Schwartz J. A. Schwartz R. B. (2010). *The Wounds that Heal: Heroism and Human Development*. Lanham, MD: University Press of America.

[B68] Singer M. F. (1991). Heroines and role models. *Science* 253 249 10.1126/science.253.5017.249 17794667

[B69] Smith G. J. (1976). An examination of the phenomenon of sports hero worship. *Can. J. Appl. Sport Sci.* 1 259–270.

[B70] Snyder M. (1984). When belief creates reality. *Adv. Exp. Soc. Psychol.* 18 248–305 10.1016/S0065-2601(08)60146-X

[B71] Sorel G. (1912). *Reflections on Violence* ed. Hulme T. E. (New York, NY: B. W. Huebsch).

[B72] Sternberg R. J. (1985). Implicit theories of intelligence, creativity, and wisdom. *J. Pers. Soc. Psychol.* 49 607–627 10.1037/0022-3514.49.3.607

[B73] Sullivan M. P. Venter A. (2005). The hero within: inclusion of heroes into the self. *Self Identity* 4 101–111 10.1080/13576500444000191

[B74] Sullivan M. P. Venter A. (2010). Defining heroes through deductive and inductive investigations. *J. Soc. Psychol.* 150 471–484 10.1080/00224540903366602 21058575

[B75] Tabachnick B. G. Fidell L. S. (2007). *Using Multivariate Statistics* 5th Edn Boston, MA: Allyn and Bacon.

[B76] Taylor S. E. Lobel M. (1989). Social comparison activity under threat: downward evaluation and upward contacts. *Psychol. Rev.* 96 569–575 10.1037//0033-295X.96.4.569 2678204

[B77] Thrash T. M. Elliot A. J. Maruskin L. A. Cassidy S. (2010). Inspiration and the promotion of well-being: tests of causality and mediation. *J. Pers. Soc. Psychol.* 98 488–506 10.1037/a0017906 20175626

[B78] Trope Y. Liberman N. (2010). Construal-level theory of psychological distance. *Psychol. Rev.* 117 440–463 10.1037/a0018963 20438233 PMC3152826

[B79] Trope Y. Neter E. (1994). Reconciling competing motives in self-evaluation: the role of self-control in feedback seeking. *J. Pers. Soc. Psychol.* 66 646–657 10.1037//0022-3514.66.4.646 8189345

[B80] Van den Bos K. (2009). Making sense of life: the existential self trying to deal with personal uncertainty. *Psychol. Inquiry* 20 197–217 10.1080/10478400903333411

[B81] Walker L. J. Frimer J. A. (2007). Moral personality of brave and caring exemplars. *J. Pers. Soc. Psychol.* 93 845–860 10.1037/0022-3514.93.5.845 17983304

[B82] Ybarra O. (1999). Misanthropic person memory when the need to self-enhance is absent. *Personal. Soc. Psychol. Bull.* 25 261–269 10.1177/0146167299025002011

[B83] Zimbardo P. (2007). *The Lucifer Effect.* New York, NY: Random House.

